# Difficult Clinical Encounters in Chronic Wound Care: An Interview Study of Nurses' Experiences and Approaches

**DOI:** 10.1111/iwj.71080

**Published:** 2026-10-27

**Authors:** Benedikt Hackert, Ulrich Weger, Ewa Klara Stürmer

**Affiliations:** ^1^ Department of Psychology and Psychotherapy University of Witten/Herdecke Witten Germany; ^2^ Comprehensive Wound Center, Department for Vascular Medicine University Medical Center Hamburg‐Eppendorf Hamburg Germany

**Keywords:** chronic wounds, emotional burden, interpersonal conflicts, non‐adherence, nurse–patient interaction

## Abstract

Although difficult social interaction patterns are scientifically addressed in other medical fields, it is an under‐researched topic in wound care. A knowledge gap exists pertaining to what kind of interpersonal problems occur between patients with chronic wounds, their relatives and nurses. In an exploratory interview study, we analyzed 37 interviews with nurses from different settings to explore how they perceive and approach difficult encounters with patients with chronic wounds. A content analysis conducted with MAXQDA revealed three broad themes of difficult nurse–patient interactions. First, conflicts included aggression, violence, insults, arguments, unrealistic demands, discrimination, lying, complaints, and general unfriendly behaviour. Often, nurses function as a kind of outlet for patients' annoyance. Second, non‐adherence was associated with perceiving treatment as difficult. Third, difficult but non‐conflictual situations were also common, that is, various and multilayered issues which complicate treatment. In addition, an overview of what respondents considered an ideal wound care practitioner is provided. Interventions could target self‐care, nonviolent communication, and health literacy.

## Contribution of the Paper

- What is already known about the topic
  ○ The health‐care setting is often a challenging environment for both providers and patients.
  ○ Difficult clinical encounters have been a research topic in a variety of medical disciplines but not in chronic wound care.
- What this paper adds
  ○ What kinds of difficult clinical encounters exist in chronic wound care, and what impact do they have on nurses' well‐being.
  ○ Non‐adherence adds to the complexity of difficult interactions between patients and health care professionals.

The health‐care setting is often perceived as a challenging environment to be involved in—from the provider‐side (i.e., as a working‐context) as well as from the recipient‐side (i.e., as a patient‐context). Difficult patient encounters are defined as ‘encounters with patients that cause strong negative emotions’ in health care professionals [1, p. 1843]. The term difficult patient is perhaps a legitimate characterization, given the frequent challenges that patients with complex backgrounds and with multifaceted problems bring to the encounter. However, we think such labels are not helpful because they place the burden of blame one‐sidedly on one end of the spectrum. In the current context, we hence prefer the term of (DCEs) which is also used in the literature [2]. Many reports of DCEs entail interpersonal conflict episodes, that is, face‐to‐face interactions encompassing, among other things, disagreement about treatment and discrepant expectations regarding the healing prospects; dysfunctional (and asymmetric perceptions of) communication including misinformation or even distrust, [3, 4, 5]; demanding, clingy, manipulative, or self‐destructive behaviour [6, 7, 8, 9, 10]; anger, agitation and (verbal) aggression [11]; and discrimination and harassment [12, 13]. Bailey et al. [14] described several physician as well as patient factors which contribute to DCEs. On the provider side, stress and exhaustion play a role, and so does a gradual cooling down due to a lifetime of commitment and regular overwork [15]. On the patient's side, psychiatric disorders such as depression or anxiety, secondary benefits from the illnesses, for example, due to monetary compensation or work‐reduction, or otherwise aggravating or disruptive behaviours are of relevance [8, 9, 10, 16, 17]. A third category of variables that contribute to DCEs includes situational factors such as time constraints due to low staffing ratios and an ensuing overload with tasks, a disagreeing third party (family members, other members of medical staff), or a difficult topic to be discussed (e.g., end‐of‐life discussions; [18]). In addition to conflict episodes, non‐adherence can be frustrating for health care professionals and is connected to perceiving patients as difficult [8, 14, 19, 20, 21, 22]. It is, for instance, an area of conflict between improving health and challenging professional control or expertise [8]. Other factors also contribute to DCEs, for example structural or systemic stressors which can affect the patient (e.g., socioeconomic situation, systemic racism, etc.) but also the health care professional (e.g., moral injury; corporatization of medicine, etc.; [14]).

Although a paucity of empirical data exists, it seems reasonable to assume that such difficulties extend to the chronic wound‐care context as well: It involves a patient population under increased health‐related pressures and with age‐specific secondary complications that contribute to difficult encounters. Like the general healthcare context, the treatment of chronic wounds is accompanied by constraints on the side of the physician, the patient and the work context: A high caseload often produces a task‐ rather than person‐centred care [23]; patients often show (non‐)intentional non‐adherence [24]; and chronic wounds, like many other forms of ailments, are moderated by a host of psychological variables that interact according to the specific constellation of (personal) factors involved. In this study, we investigated how nurses perceive and approach difficult encounters in the wound‐care context. Our goal was to provide an overview of the perceived challenges that are particularly prevalent in this type of context. To do so, we explored the variety of DCEs and patient characteristics that are seen as difficult. We also investigated how such DCEs affect nurses and how they deal with them. In addition, we identified what characterizes an ideal wound care practitioner.

## 1 Method

### 1.1 Participants

Included were health care professionals who have or had work‐related contact with patients with chronic wounds, predominantly nurses and wound care experts (i.e., nurses with additional formal training in wound care). In total, 130 participants took part in the study. Of these, twenty‐five (25 women) took part in a single interview, a further 6 (5 women) took part in a double interview and 99 (88 women, gender not identifiable for 6) took part in one of the nine group interviews. Table 1 lists the job role and type of workplace for those who took part in a single or double interview. Participants in the group interviews were nurses, MFAs (see Table 1) and certified wound experts. Workplaces included outpatient care settings, for example, general practice, home nursing service, or wound centre. The specific numbers per job role and type of workplace were not collected for group interviews due to organizational restraints, since group interviews took place as part of larger advisory boards of nurses. The size of the group interviews ranged from 6 to 17 participants. Participants were free to engage with the group interview; hence the number of participants may not equal the number of speakers in the group interviews.

**TABLE 1:** Participants' job role and workplace by interview format (single vs. double).

Interview format	Single interviews	Double interviews
	*n*	*n*
Job role		
Certified wound expert	4	3
MFA^a^	13	1
Nurse	2	—
Other	5	2
Workplace		
Clinic	18	2
Practice	2	1
Homecare	4	2
Other	1	1

^a^ A distinct health care professional in Germany who performs nursing as well as administrative tasks (MFA = medizinische Fachangestellte). It is usually considered equivalent to a registered nurse.

### 1.2 Procedure

Ethical approval for this research project was granted by the ethics committee of Witten/Herdecke University (S‐209/2022) which assured adherence to the ethical guidelines of the Declaration of Helsinki. All participants gave informed consent prior to the start of the study. Between November 2022 and December 2023, a total of 42 semi‐structured interviews were conducted. Five interviews could not be analyzed further due to interviewees not having sufficient contact with patients with chronic wounds, language barriers, or technical problems, leaving 37 interviews eligible for analysis. Single (*n* = 25) or double (*n* = 3) interviews with a duration of 10–50 min were held at different national conferences as well as at the University Medical Center Hamburg‐Eppendorf (Germany); one interview was conducted online. Group interviews (*n* = 9) with a duration of 17–44 min were conducted during advisory boards of nurses in order to receive feedback from them about their everyday wound care experiences and individual wound treatment strategies. All interviews were conducted by the first author.

### 1.3 Semi‐Structured Interviews

The semi‐structured interview guideline can be found in Appendix A. During the interviews, nurses were asked about the occurrence, experience, and handling of DCEs. Notably, we collected data not solely concerning treatment situations (e.g., dressing change) but about any form of patient contact (e.g., front desk).

The first aim was to uncover specific difficulties in the interaction between nurses and patients as well as patient characteristics that are perceived as ‘difficult’. Second, we asked how such interactions and patients are experienced, especially how they affect emotions and whether they evoke specific thoughts or trigger certain (automatic) behaviours. Third, we enquired about solution strategies: How are such situations and patients dealt with? Which strategies are constructive vs. destructive? Additionally, we asked what impact difficult interactions have on dealing with other patients or the teamwork with colleagues. Furthermore, we wanted to know skills and characteristics of an ideal wound care practitioner.

The group interviews started directly with the first thematic question: ‘When do you perceive interactions with patients as difficult?’ Single or double interviews contained a more general starter question: ‘What is your profession and role in wound care?’ In addition, we asked for specific behaviours displayed by patients with chronic wounds. Since some interviews were time‐constrained, especially the group interviews, not all questions mentioned above could be addressed in every interview. The benefit of the group interviews was that they likely encouraged discussion among participants and thus generated more input. Individual interviews, in contrast, probably created a more intimate atmosphere, which in turn made interviewees feel more comfortable revealing more details.

### 1.4 Data Analysis

The recorded interviews were automatically transcribed using Trint (Trint Limited, 2023) and then revised manually. A qualitative content analysis using MAXQDA (VERBI, 2022) was applied to all transcripts. Codes were generated using parallel inductive and deductive coding [25].

There were no preconceived categories before analysis, although the interview guideline and impressions during the interviews served as a starting point to include the different kinds of DCEs (see codes ‘conflicts’, ‘adherence’ and ‘difficult but non‐conflictual situations’ below), the effect on nurses (see code ‘stressed nurses’ below), and what makes the ‘ideal wound therapist’. The transcripts were carefully read, and any relevant text passage was either given a new (sub)code (inductive component) or assigned to an existing code (deductive component). At the beginning, most codes were inductively created from text, while at later stages deductive coding became more frequent. To reach the final category system, sub‐codes were removed or merged/split up where appropriate.

### 1.5 Positionality

The interviewer studied psychology and works as a research assistant in the field of social psychology. Except for the research project which this study is part of, he is not involved in wound care. Prior to the interviews, he had no contact with the participants or with potentially treated patients, so any impact or bias resulting from this can be ruled out. His approach to wound care is primarily theoretical in nature based on his theoretical engagement with the subject at hand. Occasional contact with wound care providers and patients was due to conference talks, data collection, or other research activities.

## 2 Results

In total, 375 codes allocated to 7 main categories (and several further sub‐categories). Figure 1 depicts the main categories and frequency of coded text segments per main code. The complete coding tree including sub‐codes is available upon request from the first author.

**FIGURE 1:**
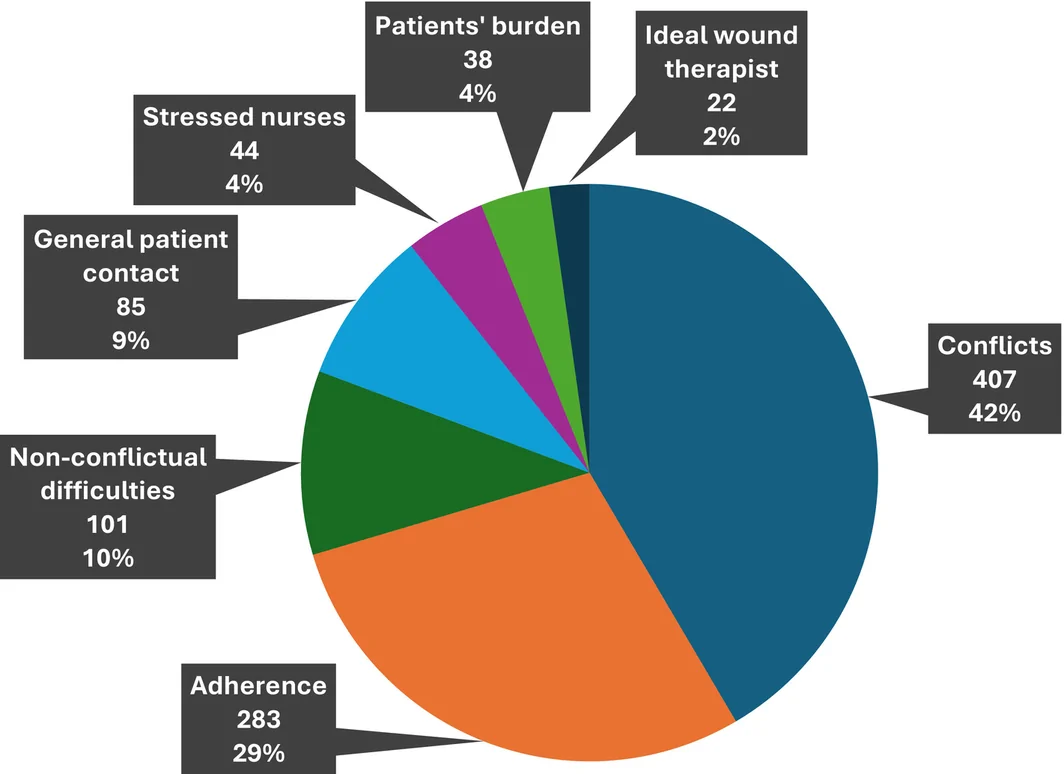
Main categories of the coding tree and frequency of coded text segments. (Numbers depict absolute and percentage frequency of coded text segment per code.)

It is important to note that the reported numbers do not indicate generalizable prevalence. For example, 42% of all coded text segments relating to conflicts does not mean that nearly half of all patient encounters are conflictual. Rather, it demonstrates that conflict is an important topic for nurses to talk about.

### 2.1 Conflicts

Concerning the frequency in everyday work, most nurses mentioned that difficult interactions are rare (20 statements stating that there are no or very few difficult interactions vs. 3 statements stating that difficult interactions are frequent) and most patients are friendly and thankful. Moreover, several nurses pointed to a dichotomy in patients: ‘Most patients are either friendly or unfriendly, there isn't really anything in between’. Nonetheless, various difficult situations were named. Most dramatically, patients can become insulting or even violent, which can make it difficult for nurses to keep a professional distance to treat all patients in the same way: ‘In that moment, he grabbed me by the collar. I told him I didn't want it, [he] insulted me and [said] “now I'm dying because of you”’. Fatphobia and racial discrimination were apparent in explicit derogatory remarks but also on a subtle level: ‘They are generally a bit cheekier with me than with my German colleague’. In addition, patients expressed the wish not to be treated by staff perceived as foreign or overweight. In addition, sexist remarks targeting the outward appearance were reported to be made mainly by older male patients.

Patients often show impatience and unfriendliness and react negatively when they are asked to help actively. Moreover, patients' reports about a previous treatment can sound dishonest or downright lying, which makes it difficult to estimate whether the reason for non‐healing lies with the applied therapy or with the patient: A patient who had to wear a special shoe which was not suitable for driving was described by one nurse as follows: ‘After arriving by car [at the wound center], he puts on [his orthopedic shoes] in the car park, limps to us …, and as soon as he leaves the wound center again, he takes it off again to drive his car’. Other instances include stating that the nursing service did not show up, or falsely and repeatedly telling someone that a silicone allergy exists. Generally, such behaviour is not interpreted as ill‐intended but as some kind of excuse to avoid arguments. One nurse told about a patient who did not let her do the wound care and put on the dressing because he perceived the handling as unhygienic—despite following standard procedure. Furthermore, some patients have unrealistic demands: a patient wanted an appointment urgently but turned down several offers and then reacted angrily when no additional ones were offered to him. Less dramatically, patients sometimes act annoyed when nurses arrive at patients' homes. In Table 2 further interpersonal problems were listed. It was assumed that inexperienced younger employees are more prone to being taken advantage of: ‘They are a ready‐made victim for some patients' frustration or sexual urges’.

**TABLE 2:** Conflicts between nurses and patients.

A visit from the nursing service is perceived as a nuisance Patient corrects or directs the nurse constantly Rude behaviours or arrogance towards nurses at the front desk Patients' non‐verbal behaviour signalling annoyance (e.g., ‘every second glance at the ceiling’) Being untalkative in the beginning when trust is not yet established Complaints (e.g., about waiting times)	Verbal (e.g., yelling) as well as physical aggression (e.g., pounding on the counter) Unfriendly or grumpy talk Raised voice Demanding behaviour (e.g., appointments, single room as privately insured patient, household help or services which are not part of the wound care [e.g., to bring out the trash]) Interrupts a conversation at the front desk Reluctant to accept standard medical procedures

### 2.2 Reasons for and Functions of Conflictual Behaviour

Assumed stressors for interpersonal conflict are complex in both their nature and seriousness (see Table 3).

**TABLE 3:** Stressors for interpersonal conflict.

No good chemistry Patient characteristics (‘those who are simply unfriendly people by nature’) Patients do not feel taken seriously Waiting times Information asymmetry (‘stupid doctors or the stupid educated people … come and talk me into my business’) A physician does not report back to the patient Long absence of a physician Enduring sustained healing process Wound pain	Disquiet Insecurity Desperation, or being overburdened (with paperwork or a big clinic) Waiting time Other health care workers were not helpful Patients consult a provider who is not appropriate Language barrier Getting non‐carbonated instead of sparkling water Quarrels or stress at home

Remarkably, nurses function as a kind of outlet for patients' annoyance. Especially at the front desk, patients target nurses, as this is ‘the first point of contact’. This can develop into risky situations: ‘He turned on me like that, hit on the counter, and that was a [catastrophe]. We were about to call the police’. In line with this, nurses must explain themselves to patients (e.g., concerning changes in clinical standards) while feeling as ‘the smallest in this whole machinery’ and still having to ‘pay for it’. In contrast to physicians, nurses and patients meet on a more horizontal power balance. Sometimes it would happen that carers are not taken seriously. Physicians who are afforded more authority are less likely to be antagonized, while patients show less hesitation towards nurses because they know that they are still responsible for their care. In addition, patients often do not address physicians directly when they are dissatisfied but complain about them when talking to nurses.

Patients' uncertainty sometimes manifests in unfriendliness or an urge to search for solutions on the internet. This creates (professional) conflicts as patients find information that does not apply to their specific situation, which can lead to misunderstandings. In addition, some patients would lose 'touch with their bodies', resulting in limited recognition of the association between lifestyle‐related risk factors (e.g., smoking and alcohol consumption) and wound development. Consequently, they may be surprised by the deterioration of the affected body part, including the emergence of open or painful wounds on the feet. These patients often react with resistance to medical advice, as they would perceive it as interference. Moreover, ‘when [a treatment procedure] is extremely restrictive, there are sometimes discussions about whether this type of therapy is the right one.’

Another reason is lack of education (see also [26]). Patients want to understand why something is being done. The need for information is significant—if they do not receive an explanation, they often react negatively. Usually, conflicts arise gradually.

Another facilitator of difficult encounters is structural in nature: The team has to move quickly from one patient to the next, which can lead to misunderstandings. Lack of time was often mentioned as a hindrance to person‐centred care: ‘And in hospital, of course. It's a big meat grinder anyway. And people don't have someone who cares for them. And if they don't talk about it at all, then of course it builds up at some point’. Another barrier to person‐centred care was the sole focus on economic profit (e.g., requesting/prescribing unnecessary expensive products).

### 2.3 Reactions to and Solutions for Conflicts

Reactions and approaches to conflicts are multifaceted (Table 4). In general, some conditions help to develop a good nurse–patient relationship. Among others, it is helpful to build up trust, to work outcome‐oriented and to deliver individual, person‐centred care (see also [27]). Moreover, to think about what we can ‘… give people to make them feel good and give them quality of life …’ can be fruitful. Nurses reported that they developed a feeling for patients over the years to know whether a patient is open or not.

**TABLE 4:** Reactions and approaches to interpersonal conflicts.

**De‐escalation** Ignore; not starting a discussion; let patients leave and bleat Professional distance and neutrality Keep being friendly; positive communication/adapt choice of words Rise above it stay calm (e.g., not raise voice in return) **Defend oneself** React unfriendly, too Reflect on one's own behaviour Stand up, tell patients it's enough, or put one's foot down Belittle the patient **Discontinue contact** Quit discussion/leave (e.g., telephone call) Another nurse or supervisor or physician takes over Quit treatment altogether Refer to specialist	**Sympathy** Empathy and understanding Encourage patient and support emotionally Listen and take patients seriously Friendly staff in general Patient‐centred care Explain, e.g., medical procedures Humour and jolly up patients Find rational explanations for patients' behaviour (e.g., bad day, sorrows) **Self‐care** Me‐time Calm oneself; wait till emotions subside No rumination afterwards To be over and done with after a treatment session Not taking it personally/distance oneself Adapt attitude, e.g., no world saviour

To clear up problems, a talk with the patient to hear about the experiences of both sides is helpful: ‘So we experience this and this as a care service, how do you see the situation?’ In this way, misunderstandings or mistakes on either side (sometimes a medical practice by another provider, that is, the general practitioner of a patient) can be resolved. Pertaining to the nurse's stress, thankful other patients restore good feelings. In addition, talking about a challenge with colleagues is of help, especially with those who also know the patient.

### 2.4 Effect on Nurses

Nurses can react differently to difficult situations. Some handle them well while others feel very stressed, which can affect their professionalism. Sometimes assignments to specific patients are even avoided. Intense situations can push nurses to their professional limits. Constant negative feedback and sole responsibility can lead to severe psychological stress and even burnout: ‘So I was on sick leave for a month. I also saw the psychologist. I was on my own the whole time and the patients were attacking me the whole time and I just couldn't take it anymore.’ Hence, unharmonious or conflictual interactions can affect nurses in negative ways. They ask themselves ‘why am I doing this?’ when patients are perceived as ungrateful.

Moreover, stressful experiences, such as the death of patients or conflicts at work, can make it difficult to remain empathetic and patient. Likewise, situations are challenging where patients do not want to co‐operate and neglect themselves and one ‘really has to abandon them to their fate’. Besides that, bad hygiene of patients can be a burden: ‘When he comes, the whole practice smells. When he leaves, we can't use the room for at least half an hour, despite ventilation and sprays and whatnot’. In an overworked practice with no support and no opportunity to vent one's burden, this can lead to nurses changing the workplace. In addition, an unfortunate treatment course can be emotionally wearing.

### 2.5 Non‐Adherence and Non‐Compliance

The project is based on the knowledge that interpersonal conflict situations are particularly common in wound care. In our interviews, we found that this was not as often the case as expected but that another common and well‐known problem bothered nearly all of the nurses interviewed: A lack of compliance and/or adherence. In Table 5, some specific compliance‐related problems can be found.

**TABLE 5:** Problems concerning adherence or compliance.

Lying Manipulation of the wound Health‐promoting behaviours perceived as too laborious Different goal than nurse Immune to feedback Use of wrong products to care for the wound	Health beliefs/patients know it better Deficits in health literacy Social needs/loneliness Self‐care behaviours are too laborious Complications concerning safety (e.g., shoes cannot be put on properly because of toe dressing, risking falling, hence dressings are not worn)

Non‐adherence in patients is often predictable during the first encounter based on facial expressions, pitch of voice, mood, impatience, patients who always have a mind of their own, or statements that orthopaedic shoes, compression, or even a plaster are not needed. Their characters are described as balky, wearisome, peculiar, immune to medical advice, resigned, in denial, passive, dependent and support‐demanding, even though they would be able to do it themselves.

The need for patient education in relation to self‐care behaviours is of utmost importance (see also [26]). A hindrance to health literacy is that patients—especially older ones—would not be interested in building knowledge about their wound. ‘You can tell them all day long how great and super it all is. They also nod along, but [as soon as I] turn around, bandages are loosened because it hurts, or they shower and soak the dressing’. Short‐term improvement occurs when suffering intensifies but wound care behaviours are stopped again when pain and other discomfort diminish.

Remarkably, relatives or even other professional caregivers were sometimes held accountable for an unrequested removal of the dressing or usage of wrong products (e.g., calendula ointment).

When asking about reasons for patients' non‐adherence, nurses reported that the problem is rarely a lack of willingness, but rather a predicament in which the patient sees no other choice. Sometimes they would lack self‐awareness to fully reflect on the situation. In addition to this, motivation for fundamental lifestyle changes was said to be lacking. Even the first consultation often happens too late, since patients seem to disregard the seriousness of their wound.

Likewise, some patients hide wounds for months and they are detected only by chance. Despite that, ironically, some patients put effort into a neat outward appearance. Assumed reasons for such different levels of care are shame and lack of knowledge about which wounds need medical care. Concerning solutions, when a goal is not achieved with a patient, it is helpful to ask directly what can be done better. Patients want to be taken seriously and have their experiences taken into account.

### 2.6 Non‐Conflictual Difficulties

It became evident that, in addition to interpersonal conflicts and non‐adherence, treatment itself can be difficult for various reasons. General factors on the patients' side are, among others, shame, fear and worries, avoidance, bad hygiene, fear of death or refusal of care.

From the interviews it became evident that such issues are multilayered and complicate the treatment. For instance, due to shame, patients would ignore the severity of a wound, leading to delayed treatment. Moreover, participants had the impression that some patients ask many questions to prolong their hospital stay, or patients with a suspected dependency syndrome would accept worsening of the wound in order to receive painkillers. Furthermore, patients are often confronted with incomprehensible technical terms, which could cause uncertainty and the feeling of being left alone. On the nurses' side there are also several complicating factors; for example, a lack of medical expertise in wound care, differing opinions pertaining to the frequency of dressing change, treating wounds located at intimate body parts, or evaluating statements of older patients or patients with dementia. Nurses reuse dressing material to save costs, which patients do not mind. However, patients often rate a wound expert who (correctly) disposes of everything negatively. Structural conditions like lack of time or several nurses caring for the same patient make patient‐oriented care difficult, and misunderstandings can occur.

### 2.7 The Ideal Wound Care Specialist

Finally, we asked what the ideal characteristics of a nurse in wound care should be. Characteristics named are depicted in Table 6.

**TABLE 6:** Characteristics of an ideal wound care specialist.

Social skills Sensitive Patient Empathetic Self‐reflective Ability to listen Communicative Flexibility Self‐confident	Positive Attitude Friendly Respectful Humorous Authentic Open‐minded Honest Openness Respect patient's pace

An ideal wound care specialist should have a good, trustful relationship with patients, consider patients' biographies, explain a lot and give reassurance. Clear and direct communication is important in order to build realistic expectations. The situation should be discussed openly, even if it is uncomfortable, and problems should be addressed. Direct feedback, even if the patient makes mistakes, is often appreciated. The nurses should show patience and convey calmness, even with patients perceived as difficult, without taking their behaviour personally. Most patients want to be actively involved, and therapy should be jointly agreed upon to promote independence, understanding and long‐term adherence—according to the definition of person‐centred care. It is crucial to explain to the patient that a wound sometimes takes longer to heal. Flexibility and openness are assumed to be important to avoid stagnation and to respond better to patients. A positive attitude helps to achieve quick results. Counselling skills are taught during training, but interpersonal skills and self‐confidence are primarily based on job experience. Wound experts need emotional distance, as the work can be stressful.

## 3 Discussion

In 37 interviews, we analyzed interactions between nurses in wound care and patients with a qualitative content analysis and identified three broad kinds of DCEs: conflicts, non‐adherence and non‐conflictual treatment difficulties. Overall, the results show that a good relationship with patients prevails in chronic wound care settings. Nevertheless, conflicts have been reported by most nurses, including aggression, violence, insults, arguments, unrealistic demands, discrimination, lying, complaints and general unfriendly behaviour. This is in line with previous studies not specifically focused on the wound care context (e.g., [18, 28, 29, 30]). Assumed stressors and reasons for conflicts are diverse in nature and seriousness. Often, nurses function as a kind of outlet for patients' annoyance; the psychological mechanism behind this might be displaced aggression [31]. Nurses with high empathy might be better equipped to interpret such behaviour ‘as a signal for needing help, rather than norm‐violating interpersonal treatment toward them’ [32]. Although this study focused on such interpersonal conflict episodes, what concerned nurses most frequently was the lack of adherence or compliance—while dyadic face‐to‐face conflicts seem to be rather rare. This contrasts with findings of a large‐scale survey among health care professionals in Germany, according to which 94.1% had experienced verbal abuse and 69.8% physical aggression in the last 12 months [28]. In addition, difficult but non‐conflictual situations were also common, leading to complications in treatment. Although different taxonomies were used, many of these themes have been reported before in the context of DCEs [18].

Reasons for DCEs have been described at various levels. At the interpersonal level, they concern primarily communication style, understanding the patient, expectations of patients and their relatives and role negotiation. In this regard, the relational character of DCEs becomes apparent. At the organizational level, imbalance of power between patients and health care professionals, availability and time per patient, or information flow between the various care providers have to be considered. At the level of institutionalization, ideological aspects such as economic profit, the discourse about patients labelled as difficult and political regulations (especially increased bureaucracy) play a role. It is noteworthy that many problems occur independently of the actual wound context or even outside treatment sessions (e.g., conflicts at the registration desk). The levels considered above fit into the classification proposed by Bailey et al. [18]. The reported consequence of difficult encounters is frustration alongside other negative thoughts and feelings, which could in turn adversely affect communication and clinical care [7, 33]. In the long run, it could lead to job dissatisfaction, reduced well‐being and burnout [34, 35]. To avoid this, it can be postulated that interdisciplinary case conferences, behavioural training and the exchange of experiences—which are often neglected due to the ever‐present time pressure—would enable the implementation of strategies to address conflictual behaviour in patient interactions, to the benefit of both parties. Moreover, stable team structures with clearly defined responsibilities and expertise, along with (personal) experience in caring for patients—who are often of advanced age and suffering from pain—appear to be advantageous.

The reports about physical or verbal abuse directed towards nurses fall into findings concerning workplace violence in health care [36, 37, 38]. Part of such behaviour can be explained by (chronic) pain [39, 40], which is often associated with chronic wounds [41]. Although abuse directed towards patients was reported only by one participant, it should be taken seriously in light of emerging research about elder abuse [42]. Poor working conditions in nursing can increase the risk of burnout which, again, could increase the risk of psychological or physical abuse [43, 44]. Since many nurses mentioned frustration and time constraints in our interviews, current circumstances in wound care pose a possible threat.

Research on medical racism has targeted both experiences of patients and health care professionals [45, 46, 47]. Only recently has such exploration in the context of chronic wounds emerged, despite ethnic/racial disparities in epidemiology and downstream complications [48]. Moreover, most studies to date have a focus on diabetes [49, 50, 51]. To the authors' knowledge, investigations into nurses' experience of discrimination are missing altogether. Therefore, the reports about racist patient behaviour and other forms of discrimination (e.g., social stigma of obesity) from our data can be taken as a starting point. Since nurses can already be strained by different stressors (e.g., wound odour, repeated patient education, non‐compliance, emotional work, wearing topics of conversation, instilling hope in patients while being realistic), coping with discriminatory talk and behaviour can further drain their well‐being [52]. Thus, effective management of such instances [53] should be of interest for anybody in the provision of treatment of chronic wounds.

Concerning health literacy, inconsistent statements pertaining to the need of it were made. Patients are said to react negatively when they do not understand enough while others would show no interest in it. This can be linked to statements about patients who seem to have lost contact with their affected body parts [54]. Hence, increasing patients' interest and education in their wounds and motivation for self‐care is imperative [55]. Concomitantly, it must be ensured that relevant information in the right amount is provided. Models of health literacy should take this into consideration.

Although DCEs should be interpreted with regard to the dynamics between provider and patient and to the wider sociological context [11, 18]—and not simply reduced to patient characteristics—some of the reported behaviours seem barely appropriate. For instance, when patients expect nurses to get them something to drink when they could easily do so themselves, or when they decline several appointment options and then react angrily when no further appointments are available. Probably this is one of the downsides of medical consumerism.

### 3.1 Limitations

As this was a qualitative, exploratory study, design‐dependent limitations exist pertaining to credibility, transferability, dependability and confirmability [56], reflexivity and subjectivity of researcher and participants [57], or social desirability bias [58]. Since ‘the interview situation itself [is] a specific context for knowledge production’ [59], the different interview formats might have affected responses. Especially in the group interviews, nurses were so eager to talk about adherence problems that it was at times challenging to redirect the interview to interpersonal conflicts. Based on the personal assessment of the interviewer, nurses were often not used to reflecting psychologically on such encounters. Descriptions were mostly pragmatic and behaviour‐focused and, consequently, some (reciprocal) psychological phenomena were potentially ignored by the nurses. In this regard, it must be highlighted that the perspective of the nurses taken in this study made a focus on problematic patient characteristics and behaviours more likely to be reported. Any of the difficult clinical encounters described above occur, of course, in an interpersonal setting where patients and nurses alike negotiate the treatment course as well as their roles and needs. Presumably, unrecognized needs might aggravate behaviour of patients perceived as a nuisance. On the other hand, ‘anger, agitation, or demanding and manipulative behaviours can elicit strong defensive responses from physicians and reduce collaborative care’ [18].

### 3.2 Future Research and Implications for Practice

Surveys to estimate the prevalence of different kinds of DCEs in chronic wound care settings are important before tailored interventions can be evaluated. Inquiring into the dynamics between macro (e.g., health care policies, nurse education), meso (e.g., discourse on ‘difficult’ patients, structural boundaries) and micro levels (e.g., communication style, patient characteristics, everyday stress) and how they affect nurse–patient relationships can be of avail to identify which are the main drivers of DCEs. Moreover, teaching self‐care, measures to promote resilience, or approaches like Name It to Tame It or CALMER could become standard in nursing student curricula [18, 60, 61]. Concerning possible physical or psychological abuse of patients as well as nurses, first it must be established how prevalent and serious such a problem is in chronic wound care settings. Subsequently, knowledge about how and when to report instances of abuse could be standardized and disseminated [62]. Future research should determine the prevalence of different difficult encounters within different chronic wound care settings (e.g., inpatient, outpatient, and community settings). To reach these goals, an interdisciplinary approach including sociological and political perspectives as well as using multiple methodologies should be most fruitful.

## 4 Conclusion

In the day‐to‐day care of patients with chronic wounds, interpersonal conflicts encompass a broad range of hostile behaviours and their stressors are diverse in nature. Nurses' responses are aimed at avoiding further escalation of the situation and maintaining their own mental well‐being, which can certainly be at risk due to conflicts. Although not inherently conflictual, non‐adherence constitutes a prominent and multifaceted issue that is closely intertwined with nurses' perceptions of patients as difficult. In addition, miscellaneous non‐conflictual difficulties may be situated at the provider, patient, or structural level. In this regard, interviewees' descriptions of an ideal wound care specialist emphasized responsiveness to patients' individual needs, reflecting the broader shift towards person‐centred care in medicine. Such an approach also carries the potential for increased patient empowerment and could be supported by the identification of patients' degrees of freedom during the treatment course [63, 64]. Future studies should take into consideration the perspective of patients with chronic wounds and how their behaviour is a reaction to factors outside the patient including nurses' behaviour, medical issues and systemic stressors.

## Funding

The presented study was funded by Dr. Ausbüttel & Co. GmbH. No direct impact on the study took place.

## Ethics Statement

Ethical approval for this research project was granted by the ethics committee of the University of Witten‐Herdecke (IRB No.: S‐209/2022) which assured following ethical guidelines of the Declaration of Helsinki.

## Conflicts of Interest

The authors declare no conflicts of interest.

## Supporting information


**Appendix A:** Semi‐structured Interview Guideline

## Data Availability

The data that support the findings of this study are available from the corresponding author upon reasonable request.
