# Supplementary material for: Difficult Clinical Encounters in Chronic Wound Care: An Interview Study of Nurses' Experiences and Approaches
Source: Int Wound J. 2026 Oct 27;23(11):e71080. doi: 10.1111/iwj.71080 (PMC13711349; doi:10.1111/iwj.71080)
Supplement: Supplementary file 1 [file IWJ-23-e71080-s001.docx]

**Appendix A: Semi-structured Interview Guideline**

*Note: This Guideline was translated into English. The original German version can be requested from the corresponding author.*

Starter Question (optional), for example: When and where do you have contact with chronic wound patients?

Part 1: Occurrence of difficult patient encounters

- When do you experience interactions with patients as difficult? What exactly happens?
  - If nurses only refer to verbal interactions, ask further: Are there any other difficulties that can occur with patients beyond pure dialogue?
- How do you recognise a ‘difficult patient’? What are patients like who you experience as difficult? How do these patients behave?

Part 2: Perception of difficult patient encounters

- How do you experience difficult interactions with patients? What does that do to you?
  - What does this trigger in you? Certain emotions or thoughts? Or a certain behaviour?

Part 3: Strategies

- How do you deal with patients who experience you as difficult? How do you react to behaviour that you find disturbing or obstructive?
- Follow-up question: Which of these behaviours/reactions have you experienced as helpful? Which ones were destructive?

Part 4: Generalizing Effects

- What effect do difficult interactions have on dealing with other patients? Or on collaboration with colleagues? Possibly also on interactions outside the immediate therapeutic care context?

Part 5: Ideal wound care specialist

- Generally speaking, what characterises the ideal wound therapist? What are their strengths? What characterises a poor wound therapist? What are their weaknesses?
